# Immunogenetic mechanisms for the coexistence of organ-specific and systemic autoimmune diseases

**DOI:** 10.1186/1740-2557-5-1

**Published:** 2008-02-15

**Authors:** Masha Fridkis-Hareli

**Affiliations:** 1 Department of Cancer Immunology & AIDS, Dana Farber Cancer Institute, 44 Binney Street, Boston, MA 02115, USA

## Abstract

**Background:**

Organ-specific autoimmune diseases affect particular targets in the body, whereas systemic diseases engage multiple organs. Both types of autoimmune diseases may coexist in the same patient, either sequentially or concurrently, sustained by the presence of autoantibodies directed against the corresponding autoantigens. Multiple factors, including those of immunological, genetic, endocrine and environmental origin, contribute to the above condition. Due to association of certain autoimmune disorders with HLA alleles, it has been intriguing to examine the immunogenetic basis for autoantigen presentation leading to the production of two or more autoantibodies, each distinctive of an organ-specific or systemic disease. This communication offers the explanation for shared autoimmunity as illustrated by organ-specific blistering diseases and the connective tissue disorders of systemic nature.

**Presentation of the hypothesis:**

Several hypothetical mechanisms implicating HLA determinants, autoantigenic peptides, T cells, and B cells have been proposed to elucidate the process by which two autoimmune diseases are induced in the same individual. One of these scenarios, based on the assumption that the patient carries two disease-susceptible HLA genes, arises when a single T cell epitope of each autoantigen recognizes its HLA protein, leading to the generation of two types of autoreactive B cells, which produce autoantibodies. Another mechanism functioning whilst an epitope derived from either autoantigen binds each of the HLA determinants, resulting in the induction of both diseases by cross-presentation. Finally, two discrete epitopes originating from the same autoantigen may interact with each of the HLA specificities, eliciting the production of both types of autoantibodies.

**Testing the hypothesis:**

Despite the lack of immediate or unequivocal experimental evidence supporting the present hypothesis, several approaches may secure a better understanding of shared autoimmunity. Among these are animal models expressing the transgenes of human disease-associated HLA determinants and T or B cell receptors, as well as *in vitro *binding studies employing purified HLA proteins, synthetic peptides, and cellular assays with antigen-presenting cells and patient's lymphocytes. Indisputably, a bioinformatics-based search for peptide motifs and the modeling of the conformation of bound autoantigenic peptides associated with their respective HLA alleles will reveal some of these important processes.

**Implications of the hypothesis:**

The elucidation of HLA-restricted immune recognition mechanisms prompting the production of two or more disease-specific autoantibodies holds significant clinical ramifications and implications for the development of more effective treatment protocols.

## Background

Autoimmune mucocutaneous blistering diseases (AMBD) such as pemphigus vulgaris (PV), pemphigus foliaceus (PF), bullous pemphigoid (BP), and mucous membrane pemphigoid (MMP), are a group of rare organ-specific diseases that affect skin and multiple mucous membranes [1-5]. PV is a potentially fatal disease characterized by the loss of intercellular adhesion of keratinocytes, resulting in acantholysis [6-8]. In the serum of PV patients, high titers of circulating autoantibodies targeting the epidermal adhesion molecule desmoglein 3 (Dsg3), one of the keratinocyte transmembrane proteins localized in the desmosome, which is essential for maintaining the integrity of the epidermis, are believed to cause clinical disease by direct binding to and disruption of desmoglein proteins [1,9]. The association of HLA antigens with the susceptibility to PV has been demonstrated in numerous studies [10-14]. It appears that PV is tightly linked to a rare haplotype HLA-DR4 (DRB1*0402) DQwB1*0302 in Ashkenazi Jews. In non-Jewish patients the haplotype is HLA-DRB1*404, DQB1*0503 [15].

Another blistering disease, MMP, which affects mucous membranes of the body, is characterized by the presence of autoantibodies to human β4 integrin [16,17], while BP which predominantly affects the skin, is associated with bullous pemphigoid antigen 1 (BPAg1) and (BPAg2) [18]. Both BP and MMP have been shown to have a strong linkage to HLA-DQB1*0301 [18,19]. It has been demonstrated that the same patient may have antibodies against more than one autoantigen within the skin and mucous membrane resulting in more than one autoimmune mucocutaneous disease. For example, patients with PF may develop BP [20,21]; patients with MMP may have PV [22], and some patients are affected with both PV and ocular cicatricial pemphigoid [23].

In contrast to organ-specific diseases, connective tissue disorders, or systemic diseases, including systemic lupus erythematosus (SLE), rheumatoid arthritis (RA), and systemic sclerosis (SSc), involve multiple tissues and organs [24-26]. Mixed connective tissue disease (MCTD) is a systemic autoimmune syndrome characterized by the presence of high titers of serum antibodies against small nuclear ribonuclearproteins (U-snRNPs) [27,28], in particular against U1 small nuclear RNP polypeptide (U1 snRNP). It has been suggested that MCTD represents a distinct clinical entity, based on clinical manifestations that separate MCTD from other connective tissue diseases [29]. Various associations of HLA antigens with MCTD have been reported, including HLA-B7 and HLA-Dw1 [30]. In another study, DR4 was found to be significantly increased in MCTD [31], whereas others reported an association between HLA-DQw3 and anti-RNP antibodies in patients with MCTD [32]. Interestingly, MCTD patients with increased IgG autoantibodies against U1–70 kD polypeptide have an increased prevalence of DR4 antigen compared with controls [33]. Furthermore, molecular biology studies have shown that most MCTD patients carrying DR4 or DR2 alleles share a region of homology consisting of seven amino acids in the DRB1 gene [34]. This "shared epitope" of DR molecules, in different alleles and in different patients with MCTD, may be important for the modulation of the autoimmune response to the U1–70 kD antigen [35]. HLA associations with organ-specific and systemic autoimmune disorders and their autoantigens are listed in Table 1.

**Table 1:** HLA associations with Mixed Connective Tissue Disease and Autoimmune Blistering Diseases

Disease	HLA allele	Autoantigen	Reference
Mixed Connective Tissue Disease	HLA-B7, HLA-Dw1 HLA-DQw3	U1 sn-RNP polypeptide U1 sn-RNP polypeptide	30 32
Mucous Membrane Pemphigoid	HLA-DQB1*0301	β4 integrin	19, 64
Bullous Pemphigoid	HLA-DQB1*0301	BPAg1 (BP180) BPAg2 (BP230)	18, 64
Pemphigus Vulgaris	HLA-DR4 (DRB1*0402), DQwB1*0302 HLA-DRB1*404, DQB1*0503	Dsg3 Dsg3	65 65

It has been well documented that autoimmune diseases may coexist in the same patient, either sequentially or concurrently [21,36-46]. PV, dermatitis herpetiformis, BP, and SLE have all been reported in association with other autoimmune diseases as well as with each other. In particular, observations of dual autoimmunity in some patients who concurrently develop organ-specific and systemic disease have been reported [38,40,41,44,46]. Multiple factors, including those of immunological, genetic, endocrine and environmental origin, contribute to the above condition. The immunogenetic mechanisms of this phenomenon present an intriguing unresolved problem of autoimmune predisposition, calling for development of prospective approaches of prediction and ultimately prevention of the disease. As a matter of fact, the involvement of T cells in immunopathogenesis of MCTD, PV and MMP has been well established. In MCTD, the role of anti-RNP-reactive T cells in autoantibody production has been demonstrated [47,48]. In PV, it has been shown that B cells function as antigen-presenting cells stimulating Dsg3-specific CD4^+ ^T helper (Th) cells to secrete cytokines such as interleukin (IL)-4, IL-6 and IL-10 which are required for proliferation of memory B cells and differentiation into antibody-producing plasma cells [49-51]. Thus, the interplay between B and T cells seems to be critical, which is further supported by the finding that depletion of CD4^+ ^T cells prevents antibody production (reviewed in ref. [52]). Moreover, a clinical study showed that the mean frequency of Th2 CD4^+ ^T cells was significantly elevated in PV patients with active disease, while no responses were detected for patients with disease in remission or controls [53]. Characterization of autoreactive T-cells has led to identification of immunodominant T-cell epitopes and the repertoire of Dsg3- or Dsg1-specific T-cells at the clonal level [54,55]. Lastly, the potential role for antigen-specific autoreactive T cells in the pathogenesis of MMP has also been addressed [56,57].

Collectively, these observations suggest that T cell epitopes of the respective autoantigens, i.e. Dsg-3 for PV, β4 integrin for MMP, and U1 snRNP for MCTD, may bind to their HLA molecules and trigger the activation of autoreactive T cells, which in turn would induce production of pathogenic autoantibodies. This mechanism would most probably be applicable to the case of a single disease, however, when two or more autoimmune diseases occur in the same patient, the molecular and cellular events are likely to be more complex. Based on the accumulated evidence of shared autoimmunity, it has been intriguing to investigate the relationship between the genetic and immunological mechanisms for the simultaneous production of two or more autoantibodies. A hypothesis, which in part may explain some of the increased susceptibility to both autoimmune blistering and systemic connective tissue diseases, is presented below.

## Presentation of the hypothesis

**Figure 1:**
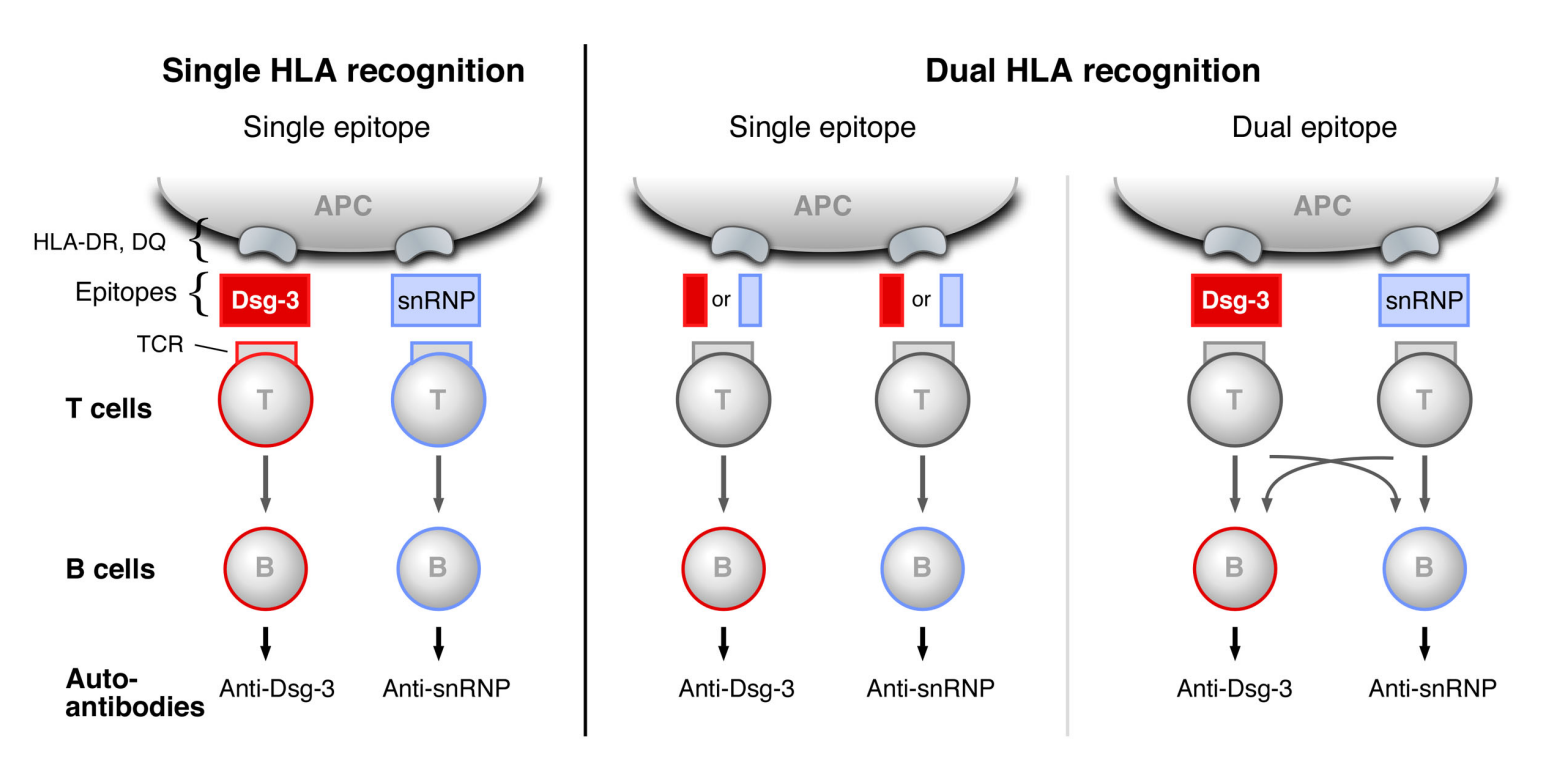
**Immunogenetic mechanisms of dual autoimmunity**. Coexistence of two autoimmune disorders in the same patient may occur due to multiple mechanisms. Schematic representation of the potential pathways leading to the induction of PV and MCTD is shown on the three panels. Based on the assumption that production of autoantibodies is triggered by T cells interacting with the autoantigenic epitopes bound to the susceptible HLA alleles, the following scenarios are described: 1 (left panel, Single HLA recognition). In this case, each T cell epitope specific for a single disease may associate with its susceptible HLA protein, leading to T cell activation and subsequent stimulation of B cells to produce autoantibodies, which would result in dual autoimmunity. 2 (central panel, Dual HLA Recognition). Here, each of the disease-specific autoantigens may bind to either HLA protein, leading to the induction of both diseases by cross-presentation. 3 (right panel, Dual HLA Recognition). According to this scenario, two distinct epitopes of the same autoantigen may be able to bind two disease-associated HLA molecules. Similar pathways would apply to the situation when MCTD and MMP are presented in the same patient.

The schematic representation of the possible immunopathogenic mechanisms leading to breakage of tolerance and induction of the two autoimmune diseases in the same individual is shown in Fig. 1. Theoretically, there can be at least three possible scenarios. The first scenario (left panel, Single HLA Recognition) may apply to the situation when T cell epitopes of the two different autoantigens associate with each of the susceptible HLA molecules, resulting in dual autoimmunity. It will occur when a single epitope of an autoantigen recognizes the disease-linked HLA determinant. For example, in the patient with MCTD and PV, carrying two DR4 alleles, one DR4 allele may bind to the relevant epitope within U1-snRNP antigen and produce anti-RNP antibodies. The second DR4 allele, such as DRB1*0402 may bind the relevant epitope of Dsg3 and result in the production of autoantibodies to Dsg3. In another instance, the DQB allele such as DQB1*0301 could present the BP or MMP antigens and thus induce antibodies to BPAg2 or human β4 integrin subunit. Based on whether the antigen presented is BPAg2 or β4 integrin subunit, the patient may have BP or MMP, respectively.

In support to this mechanism, it is noteworthy that the affinity of the binding between the autoantigenic peptide epitope, the susceptible HLA and the TCR plays an important role in T cell activation. Due to certain degree of promiscuity and specificity in peptide recognition by the HLA receptors, not a single binding affinity, but rather a range of affinities would account for the productive interaction between the peptide epitope, HLA and the TCR, leading to T cell-mediated B cell activation and antibody secretion. Of the three autoantigens mentioned in the present study, i.e. snRNP, β4 integrin and Dsg-3, the latter has been characterized most extensively in terms of epitope mapping and HLA binding capacity. Modeling of the bound conformation of PV-associated peptides revealed the role of DRB1*0402 in the selection of specific self-epitopes [58]. Several studies suggest that autoantigenic peptides do not necessarily bind to disease-associated HLA molecules with high affinity, but rather within the intermediate range, thus allowing for the rescue of autoreactive T cells. In contrast, protective HLA proteins are more efficient binders of self-antigens, which results in elimination of autoreactive T cells [58].

The second scenario (Figure 1, central panel, Dual HLA Recognition) applies to the situation when a single epitope of an autoantigen binds to both HLA specificities, leading to the induction of both diseases by cross-presentation and reactivity. Thus, it can not be excluded that there is cross-reactivity in the binding of immunopathogenic epitopes to either of the susceptible genes in the same individual, leading to T cell responses which trigger autoantibody production. For example, if an epitope within Dsg3, β4 integrin, BPAg2, or U1-snRNP is available for antigen presentation, it is possible that such a peptide with the complete homology is present in all four molecules or only in one molecule. However this specific epitope might have the capacity to bind to different HLA determinants. If it binds to DR2 or DR4, it stimulates T cells that induce activation of B cells producing anti-RNP antibodies. If it binds to DRB1*0402 or DQB1*0503, it results in stimulating T cells inducing production of anti-Dsg3 antibodies. If it binds to DQB1*0301, it stimulates T cells leading to generation of autoantibodies to BPAg2 or β4 integrin.

It should be noted that peptide binding to HLA is facilitated by the interactions between the amino acid residues lining the groove of HLA molecules and the side chains of the bound peptide. The binding pockets of HLA class II, defined by the polymorphic β chain and the more conservative α chain of the αβ heterodimer, share homology between some alleles but may also differ from other alleles as defined by size, charge and hydrophobicity [59]. Thus, peptides derived from different autoantigens may not share sequence homology, but still be able to bind different HLA due to the presence of certain amino acids which would fit to the binding pockets of the HLA molecules. Given that the sequences of Dsg-3, snRNP and β4 integrin are all different, it is unlikely that the immunodominant epitopes of each of these antigens would contain similar amino acid sequence. In spite of this fact, the two peptides may share common binding motifs, dictated by structural requirements of the HLA pockets accommodating the peptides. Due to the degenerate nature of the HLA binding and TCR recognition, the observation which has been widely accepted for the past decade [60], common binding motifs would be sufficient to allow peptide binding to the same HLA molecule. However, in this case, it is possible that the recognition of the HLA/peptide complex by T cells will differ depending on the orientation of the TCR interacting with the amino acids facing away from the binding groove, and thus will result in differential activation by T cells.

The third scenario is presented on the right panel of Figure 1 (Dual HLA Recognition). It is conceivable that two distinct epitopes of the same autoantigen are able to bind two HLA specificities associated with the two diseases, implying that both T cell epitopes originating from one autoantigen will activate immunopatogenic mechanisms by binding to two HLA molecules specific for two diseases. For instance, the patient has a single autoantigen such as Dsg3 or U1-snRNP. One epitope within this molecule may bind to the DR4 or DR2 allele associated with MCTD, resulting in anti-RNP antibody production. This would presume that certain sequences within Dsg3 are similar to sequences within U1sn-RNP. A second epitope within the same molecule may bind to the other DR/DQ allele which has the genotype associated with PV, i.e. DRB1*0402 or DQB1*0503, and produce antibodies to Dsg3. Hence, there is no cross reactivity between the two epitopes within the same molecule. However, the autoantibodies produced are different because the alleles to which they bind are different, yet specific for each disease. In this respect, it is of notion that epitopes derived from a single antigen may be generated in the course of antigen processing and presentation. Since these peptides differ in amino acid sequence and most probably bind HLA with differential affinities, it is possible that T cells interacting with the APC may also have different specificities. Thus, it is not excluded that some of these peptides, derived from one antigen, will have the potential to trigger T cells specific for the second antigen by virtue of cross-reactivity. Consequently, distinct B cell clones will be activated and induced to secrete each a different autoantibody. In this case, B cells may also serve as the APC.

## Testing the hypothesis

Molecular and cellular mechanisms governing concurrent or sequential presence of autoimmune blistering and systemic diseases in patients remain to be elucidated. Although there is no immediate or unequivocal experimental evidence to support the present hypothesis, several approaches might hold a promise for the better understanding of the shared autoimmunity. Investigation of these mechanisms has been significantly delayed due to the lack of animal models in which both the systemic and organ-specific autoimmune diseases can be induced. To this end, only a small number of experimental models of susceptibility to a single disease have been developed with limited success [61-63]. Development of such animal models allowing investigation of the effects of the triggering factors on shared autoimmunity would require genetic manipulations enabling to introduce the elements of susceptibility, i.e. human HLA and/or autoantigen-specific TCR/BCR. Thus, a transgenic mouse model expressing two disease-associated HLA and two TCR/BCR specific for each of the autoantigenic peptides would be most suitable for this purpose. In these mice, the experimental approach would contain the administration of disease-inducing peptides, separately or concomitantly, and monitoring the animals for manifestations of each disease. In parallel, ex-vivo functional analysis including antigen-specific proliferation, cytokine secretion and antibody phenotyping, has to be performed. The *in vitro *binding studies employing purified HLA proteins and synthetic peptides, and the cellular assays with antigen-presenting cells and patient's lymphocytes would also be instrumental. Undoubtedly, bioinformatics-based search for peptide motifs and the modeling of the bound conformation of the autoantigenic peptides associated with the respective HLA alleles will shed light on some of these important processes.

## Implications of the hypothesis

The hypothesis presented above could partially explain the simultaneous production of two or more pathogenic antibodies in patients having a blistering and a systemic autoimmune disease, as reported over the past decades [21,36-46]. Further studies of these patients, and especially of T and B lymphocytes administered into HLA-transgenic mice, will provide valuable information on cellular and molecular mechanisms critical for immunoregulation and production of pathogenic autoantibodies. Such studies have significant clinical ramifications and implications for the development of novel immune therapies targeting both autoimmune diseases.

## Abbreviations

AMBD, autoimmune mucocutaneous blistering diseases; APC, antigen-presenting cells; Dsg, desmoglein; HLA, human leukocyte antigens; IL, interleukin; MCTD, mixed connective tissue disease; MMP, mucous membrane pemphigoid; PV, pemphigus vulgaris; RNP, ribonucleoprotein antigen; TCR, T cell receptor; Th, T helper cells.

## Competing interests

The author(s) declare that they have no competing interests.
